# Bioinformatics-Based Screening Approach for the Identification and Characterization of Lipolytic Enzymes from the Marine Diatom *Phaeodactylum tricornutum*

**DOI:** 10.3390/md21020125

**Published:** 2023-02-14

**Authors:** Victor Murison, Josiane Hérault, Benoît Schoefs, Justine Marchand, Lionel Ulmann

**Affiliations:** 1 BiOSSE, Biology of Organisms: Stress, Health, Environment, Département Génie Biologique, Institut Universitaire de Technologie, Le Mans Université, F-53020 Laval, France; 2 BiOSSE, Biology of Organisms: Stress, Health, Environment, UFR Sciences et Techniques, Le Mans Université, F-72085 Le Mans, France

**Keywords:** diatom, *Phaeodactylum tricornutum*, lipase, transcriptomics, nitrogen starvation

## Abstract

Oleaginous diatoms accumulate lipids of biotechnological interest when exposed to nutrient stress conditions such as nitrogen starvation. While accumulation mechanisms are well-known and have been engineered to improve lipid production, degradation mechanisms remain poorly investigated in diatoms. Identifying lipid-degrading enzymes is the initial step to understanding the catabolic processes. In this study, an in silico screening of the genome of *Phaeodactylum tricornutum* led to the identification of 57 putative triacylglycerol lipases (EC 3.1.1.3) grouped in 4 families. Further analysis revealed the presence of conserved domains and catalytic residues of lipases. Physico-chemical characteristics and subcellular localization predictions highlighted that a majority of these putative proteins are hydrophilic and cytosolic, suggesting they could be recruited to lipid droplets directly from the cytosol. Among the 57 identified putative proteins, three lipases were identified as possibly involved in lipophagy due to a potential vacuolar localization. The expression of the mRNA corresponding to the 57 proteins was then searched in 3 transcriptomic datasets obtained under nitrogen starvation. Nine genes were highly regulated and were considered as encoding enzymes with a probable important function in lipid catabolism. A tertiary structure prediction of these nine candidates yielded eight functional 3D models. Among those, two downregulated enzymes, Phatr3_J54974 and Phatr3_EG00720, were highlighted as good targets for future functional genomics and purification studies to investigate their role in lipid degradation.

## 1. Introduction

Diatoms form a group of eukaryotic microalgae found in all ecosystems, including the narrowest ones [1,2]. Diatoms contribute to around 20% of global carbon fixation [3,4]. During a nutrient stress, especially a nitrogen starvation or limitation, diatoms accumulate storage compounds [5,6]. Some oleaginous species can accumulate up to 60% of their biomass as triacylglycerol (TAG) [7]. Moreover, these species can reach high biomass productivity. Hence, diatoms can be considered as a promising source of biodiesel [8]. Additionally, diatom lipid profiles are rich in omega-3 long-chain polyunsaturated fatty acids, especially eicosapentaenoic acid (EPA), essential components of human nutrition with beneficial properties in the development of infants and for a good cardiovascular health [9].

Produced TAG under stress are stored in specific compartments, namely lipid droplets (LD). LD consists in a hydrophobic core made of TAG enclosed in a monolayer membrane of polar lipids associated with proteins [10,11]. The metabolic pathways leading to an accumulation of lipids have been extensively investigated in the diatom model *Phaeodactylum tricornutum*, using several strategies including the exposure to nutrient deficiencies [12,13], the addition of inorganic and organic carbon in the medium [14,15], changes in the light regime [16,17], or the use functional genomics tools. The latter studies led to strains with enhanced lipid production and/or content in EPA. Two main strategies have been followed: (1) the “pull” strategy where fixed carbon is directed towards TAG and/or EPA by overexpressing enzymes encoding rate-limiting steps in the biosynthetic pathways [18,19,20,21,22,23], and (2) the “push” strategy where the production of precursors (including fatty acids, acetyl-CoA and reducing equivalents) is enhanced by overexpressing enzymes such as the malic enzymes [24,25,26], or by inactivating other precursor consuming pathways such as the production of sterols [27] or of polysaccharides [28,29]. A third strategy can be used to produce high lipid strains: suppress the processes that degrade lipids (“protect” strategy) [30]. Two lipid catabolic pathways are supposed to be active and both involve lipolytic enzymes: lipolysis and lipophagy [31,32]. Lipolysis occurs at the membranes of LDs where lipolytic enzymes can access the hydrophobic core and produce fatty acids and glycerol by hydrolysis. Lipophagy occurs through the process of autophagy. LDs are transported to the vacuole where specific enzymes functioning in acidic conditions degrade them. Among lipolytic enzymes, TAG lipases (EC 3.1.1.3) are able to hydrolyze the ester bond that links medium to long chain fatty acids to the glycerol backbone of TAG [33]. TAG lipases belong to the α/β hydrolases, a family of enzymes characterized by a specific fold consisting of one β sheet surrounded by two layers of α helixes, and the presence of a GXSXG pentapeptide motif [34]. Some patatin-like phospholipases and GDSL lipases, which are not α/β hydrolases, may also degrade TAG [35,36,37]. Patatin-like phospholipases include the GXSXG motif and often have a phospholipase A2 activity (EC 3.1.1.4) while GDSL lipases are characterized by a distinct highly conserved *N*-terminal GDS(L) motif and can have various activities such as arylesterase (EC 3.1.1.2) or lysophospholipase (EC 3.1.1.5) [38].

Among the 49 proteins annotated as lipases in the genome of *P. tricornutum*, only 3 have been studied by functional genomics approaches. These three proteins are reported as a patatin-like lipase (tgl1, Phatr3_J1971) [30], a plastid localized lipase (OmTGL, Phatr3_J37711) [39] and a homolog of the human CGI-58 lipase activator known to have a TAG lipase activity in diatoms (Phatr3_J54974) [40,41]. In these studies, a knockdown of the lipase gene led to an increase in lipid accumulation while slightly affecting the cell density reached at the stationary phase, either by enhancing (+17%), or by decreasing it (−6 to −10%). Additional studies have been conducted on other diatoms with the knockdown of a CGI-58 homolog in *Thalassiosira pseudonana* [42] and of a tgl1 homolog in *Fistulifera solaris* [43], both showing an increase in lipid accumulation while not affecting division rate [42] or even enhancing it [43]. This inactivation approach therefore allows to obtain strains displaying a high lipid productivity. An overexpression of certain lipases may also unexpectedly lead to an enhanced production of neutral lipids, as recently observed with the CGI-58 homolog overexpression in *P. tricornutum* (Phatr3_J54974), suggesting a central and complex role of this protein in the regulation of lipid homeostasis [41]. Moreover, the high number of uncharacterized lipases in *P. tricornutum* could also be a source for the discovery of biotechnologically relevant enzymes, as lipases are widely used in industrial processes [44].

The above-described studies have all focused on one protein at a time to highlight their eventual functional role. However, such an approach does not provide a global view of all the potentially active lipases. In the present study, an in silico screening analysis of the *P. tricornutum* genome and transcriptome has been conducted to identify a complete set of the lipolytic enzymes participating in lipid degradation processes. This work aims at elucidating key enzymes in the lipid catabolism response of *P. tricornutum* to changes in nitrate availability, a necessary first step to enhance lipid production by functional genomics. Our main working hypothesis to look for functionally relevant enzymes is that highly regulated proteins during a nitrogen starvation are also involved in the response to a nitrogen resupply with an opposite regulation, as previously observed for lipases in other microalgae [45,46,47]. Thus, in this work, candidate lipase protein sequences were retrieved from the genome of *P. tricornutum* and used for predicting their 3D conformation, physico-chemical characteristics as well as their cellular localization(s). Their involvement in lipid homeostasis is discussed on the basis of transcriptomic data.

## 2. Results and Discussion

### 2.1. Retrieval of Sequences and Annotation Screening Confirm the Existence of a High Number of Putative Lipases in P. tricornutum

The search of the keywords “lipase”, “phospholipase” and “alpha/beta hydrolase” in the Phatr3 annotation of the *P. tricornutum* genome [48] yielded 162 putative protein sequences, of which 90 have a significant annotation (E-value < 10^−5^) in the pfam database. Additional screenings through sequence investigation and ESTHER database homology search narrowed the total to 74 putative protein sequences (Table S1). Among them, 58 proteins with a potential TAG lipase function grouped in four families annotated as α/β hydrolase (24 proteins), class 3 lipases (18 proteins), GDSL-like lipases (8 proteins) and patatin-like phospholipases (8 proteins). Sequence alignment was performed to retrieve conserved motifs.

### 2.2. Multiple Sequence Alignment Allows the Identification of Conserved Domains and Residues

**Figure 1:**
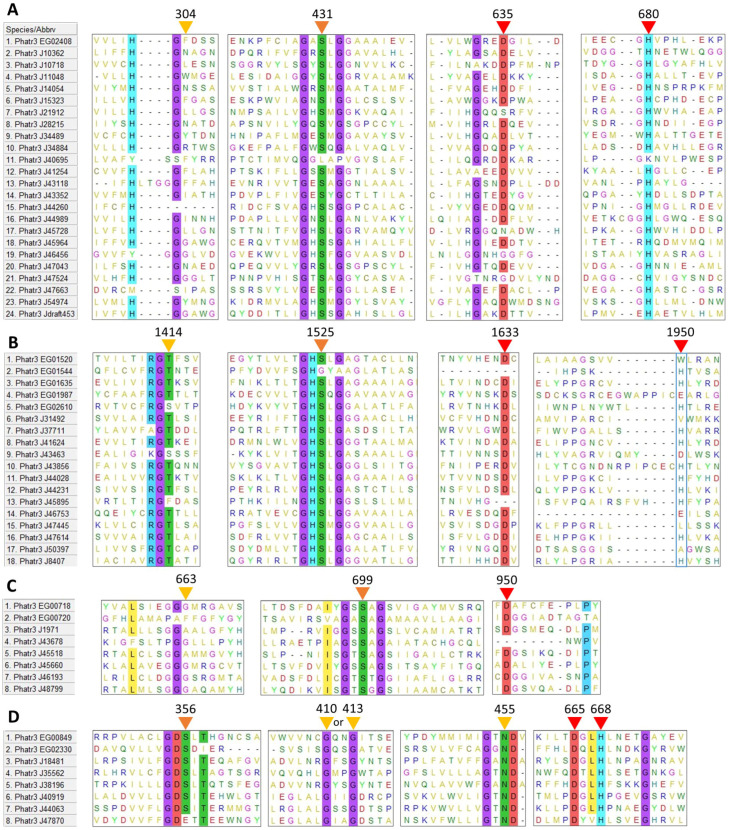
Multiple sequence alignment of putative amino acid sequences of lipases identified in the genome of *P. tricornutum*. (**A**): α/β hydrolases, (**B**): Class 3 lipases, (**C**): Patatin-like lipases, (**D**): GDSL lipases. Positions of possible residues of interest are indicated with triangles: yellow for oxyanion hole residues, orange for the catalytic serine, red for other catalytic residues. Residues conserved in more than 80% of the sequences are highlighted. Residue positions are only indicative as they include gaps created by the process of alignment.

Among amino acid residues present in the catalytic site, the activity of TAG lipases, including GDSL lipases, is due to three residues: a serine (S), an acidic amino acid (aspartic or glutamic acid, D or E) and a histidine (H). The reaction center of patatin-like lipases requires only two active residues, the serine and the acidic amino acid [49]. The multiple sequence alignment of each family revealed that possible catalytic residues are localized at mostly conserved positions across sequences (Figure 1). The acidic residue and histidine are found at isolated positions (not in a conserved motif) in α/β hydrolases (635 and 680 respectively, Figure 1A), class 3 lipases (1633 and 1950, Figure 1B) and patatin-like lipases (950 acidic residue, Figure 1C). In GDSL lipases (Figure 1D), these residues are found within a conserved motif (665 and 668 respectively), as expected for this family of proteins [38]. Additionally, the catalytic histidine position of class 3 lipases does not appear to be highly conserved (1950, Figure 1B).

After inspection of the protein sequences, in all the four retained families, the catalytic serine was always found located within one characteristic domain, either a GDS(L) motif belonging to the *N*-terminal domain, or a central GX_1_SX_2_G pentapeptide motif where X_1_ and X_2_ are variable amino acids for α/β hydrolases, class 3 lipases and patatin-like lipases.

For the class 3 lipase family, GHSLG (1523, Figure 1B) is the most common pentapeptide motif, as already observed for lipases from fungi (e.g., *Rhizomucor miehei*) [50], from the green microalga *Chlorella* sp. [51] and from the diatom *Fistulifera solaris* [52]. 

For α/β hydrolases, amino acids at positions X_1_ and X_2_ are variable: at X_1_, the most common residue is a histidine representing 33% of the sequences, while at X_2_ a majority of sequences displays a hydrophobic residue (I, L, M, V, A). For patatin-like phospholipases, X_1_ is either A, S or T while X_2_ is A, T or G which is also common for this class of proteins [35,53]. Based on the alignment, two sequences, the α/β hydrolase Phatr3_J40695 and the class 3 Phatr3_EG01544 did not seem to contain the active pentapeptide, however, closer inspection of their sequence revealed that they contained respectively a GVSHG and a GRSTG pentapeptide motif (not apparent in Figure 1). Hence, they were kept in the dataset. 

For GDSL lipases, the *N*-terminal motif (GDS at position 354) is conserved across sequences, except for Phatr3_J47870 which contained a GDE motif lacking the catalytic serine (Figure 1D). It is hence probably inactive and was excluded from further analysis. Thus, only 57 proteins of the 58 initially retained were subjected to physico-chemical characteristics prediction (see Section 2.3).

Besides catalytic residues, other amino acids are necessary for the activity of lipolytic enzymes. During TAG cleavage, reaction intermediates are stabilized by hydrogen bonds with the backbone of two amino acids which form a structure named the oxyanion hole. One of these two amino acids is the X_2_ residue in the conserved pentapeptide motif, while the second one is reported to be located in the *N*-terminal region of the protein. This second residue has been used to define three main classes of lipases: GX, GGGX and Y [33] where the most commonly found GX and GGGX types. In our work it can be inferred that all α/β hydrolases and class 3 lipases belong to the GX type, where X is the active residue (304, Figure 1A; 1414, Figure 1B). Patatin-like phospholipases display a motif resembling GGGX type (Figure 1C). We observed that X is mainly T (1414, Figure 1B) in class 3 lipases, as previously described in filamentous fungi lipases [54]. For GDSL lipases (Figure 1D), a distinct oxyanion hole motif is formed by the catalytic serine, associated with a conserved glycine and asparagine in two distinct sequence blocks as previously reported [33].

In lipases containing the pentapeptide motif, the type of oxyanion hole is a factor of substrate specificity: GX type lipases usually hydrolyze medium and long carbon chains, while GGGX type is more specific towards short length substrates [54]. Indeed, the fatty acid profile of *P. tricornutum* TAG is mainly composed of medium and long-chain fatty acids (C14:0, C16:0 and C16:1) [55] which is in line with GX type lipases that could degrade them. For patatin-like phospholipases, even though their oxyanion hole motif resembles a GGGX type, some homologs from different microalgae have been shown to hydrolyze TAG with long to very long chain fatty acids [30,43,45,56].

### 2.3. Physico-Chemical Characteristics Provide General Information about Candidate Lipolytic Enzymes and Insigths of In Vivo Function

**Figure 2:**
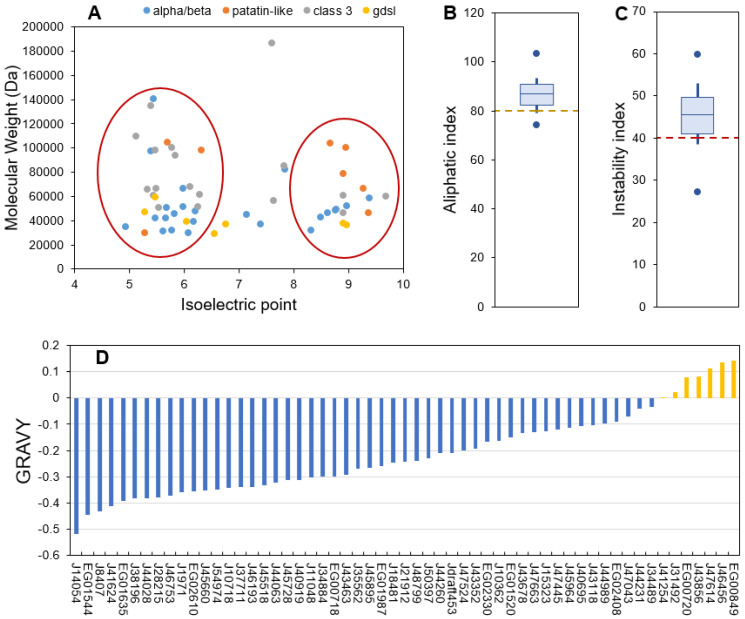
ProtParam calculated physico-chemical characteristics of the candidate lipases. (**A**): molecular weight as a function of isoelectric point, (**B**): aliphatic index, (**C**): instability index, (**D**): Grand Average of Hydropathy (GRAVY) score). For aliphatic and instability indexes boxplots, the central bar indicates the median, boxes indicate first and third quartiles, error bars indicate first and ninth deciles, and endpoints indicate maximum and minimum values.

The ProtParam server provided a range of parameters for predicting the different physico-chemical properties of the 57 candidate proteins (Table S2). The length of the sequences ranged from 253 to 1709 amino acid residues (molecular weights (MW) from 28.8 to 186 kDa) (Figure 2A). Isoelectric points (pI) ranged from 4.94 to 9.68 (Figure 2A). These values are compatible with the global range of *P. tricornutum* proteins as obtained from proteomic data which MW goes from 10–150 kDa (with a few outliers bigger than 150 kDa) and pI from 2 to 10 [57]. Lipases from bacteria and fungi are reported to have MW going from 19 to 60 kDa [44]. The higher MW obtained for *P. tricornutum* lipases could be explained by longer sequences with additional domains such as transmembrane domains, as reported for *Chlorella* sp. [51]. Independently of their families, the candidate lipases can also be separated into two major pI groups: an acidic one ranging from 5 to 6 and a basic one ranging from 8 to 10 (Figure 2A). A pI bimodal distribution that excludes the physiological pH of 7.5 is a common feature of proteomes from all domains of life and is expected as proteins are usually less soluble and reactive in a pH close to their pI [58]. Both MW and pI are useful characteristics to identify enzymes during a purification process.

The aliphatic index is an indicator of the volume occupied by aliphatic amino acid residues (alanine, isoleucine, leucine and valine) which provides an indication of thermal stability. Proteins with a proven thermostable character have an index value higher than 80 [59]. In candidate lipases, the aliphatic index ranged from 74.05 to 103.19, suggesting that most proteins are thermally stable which is an interesting feature for isolation and purification (Figure 2B).

The instability index is an estimator of in vivo stability of a protein, often used for estimating in vitro stability [60,61]. Proteins above an instability index of 40 are considered unstable, which means that their in vivo half-life is lower than 5 h, while stable proteins have an instability index under 40 and a half-life higher than 16 h [61]. Independently of their families, 75% of the candidate proteins were predicted to have a short in vivo half-life (Figure 2C). This feature is coherent with a function as stress-responsive candidate lipases that would be produced only for a transitory lipid degradation process. Indeed, a short half-life is common for proteins involved in the response to perturbations as a rapid degradation is necessary to induce changes in protein abundance that allow a metabolic response [62].

The GRAVY score is an indicator of protein hydrophobicity or hydrophilicity, this score being positive for hydrophobic proteins [63]. Here the GRAVY scores ranged from −0.52 to 0.143 with a majority of hydrophilic proteins (Figure 2D). Only six proteins displayed a slight average hydrophobic character, including members of the four described families (see Section 2.1). Lipases are generally interfacial proteins, meaning that their activity is maximal when they are located at the interface between water and a lipidic compartment or membranes [33], hence they have both lipid-interacting hydrophobic domains and more abundant hydrophilic ones that permit storage in the cytosol. The interfacial character of lipases permits the hydrolysis of TAG stored in lipid droplets (LDs). LDs are dynamic compartments surrounded by a single layer membrane made of phospholipids and proteins. Two classes of LD proteins have been defined based on association dynamics: class I proteins are embedded in the LD membrane through a hairpin domain and are located at the Endoplasmic Reticulum (ER) membranes in the absence of LDs, while class II proteins are recruited to the LD surface directly from the cytosol [64]. The general hydrophilic character of the analyzed proteins suggests that they cannot be permanent residents in lipidic compartments and are thus probably class II proteins.

### 2.4. Predicted Subcellular Localization Varies across Families and Is Mainly Cytosolic

**Figure 3:**
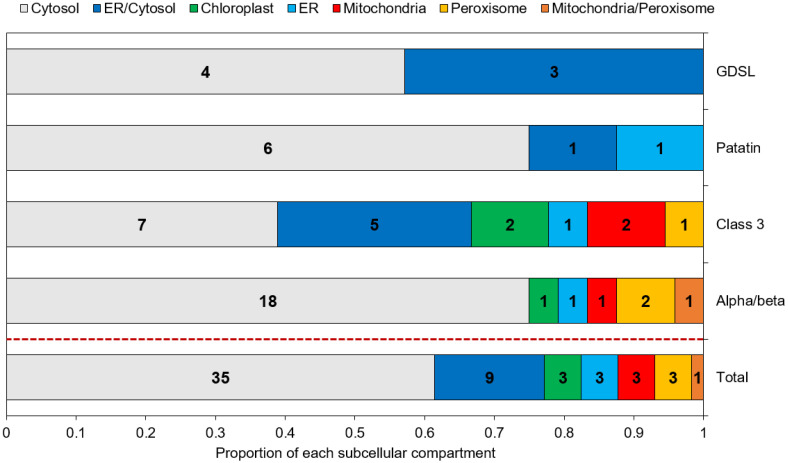
Predicted subcellular localization of the candidate lipases. Numbers inside each box indicate the number of proteins assigned to the corresponding compartment.

Subcellular localization is an important parameter to determine the in vivo function of proteins. Predictions obtained through a combination of various freewares are displayed in Figure 3 and Table S3. Overall, 35 (61%) of the putative lipases are predicted to be cytosolic, and 9 (16%) are predicted as cytosolic or associated to the ER, as they contain at least one predicted transmembrane domain. The remaining proteins (23%) are assigned to the chloroplast, ER, mitochondria and peroxisomes. Patatin-like and GDSL lipases are all assigned to the cytosol and/or the ER. Proteins of the α/β hydrolase family and class 3 lipases were located in all the possible individualized compartments.

In various species, lipases have been shown to be co-located with LDs, permitting direct lipolysis as it is the case for the human adipose triglyceride lipase [36], for the sugar-dependant-1 (SDP-1) lipase in germinating seeds of *Arabidopsis* [35], or for two TAG lipase of the green alga *Chromochloris zofingiensis* expressed in yeast cells [65]. However, no tool is currently able to predict a localization to lipid droplets. Hence, such a prediction can only rely on previous studies and known addressing mechanisms.

Four studies aiming at determining the LD proteome of *P. tricornutum* have been conducted, using a nitrogen starvation as the stress condition permitting an accumulation of TAG and the subsequent formation of LDs [10,11,66,67]. In these proteomic datasets, except for one α/β hydrolase, Phatr3_J43352 which was found in the LD proteome of *P. tricornutum* Pt4 strain [10], no annotated TAG lipase has been reported. This result is in line with our previous assumption of candidates being class II LD-associated proteins based on their general hydrophilic character and their cytosolic subcellular localization.

As almost no lipase was found in LD proteomes, lipases could be produced and translocated to LDs only in situations where TAG are degraded. Indeed, it was observed in different species of microalgae, that nitrogen resupply acts as a switch for the expression of TAG lipase genes [45,46,47]. Lipases can also be produced during lipid accumulation and being translocated to LDs once nitrogen is resupplied. Such a scenario occurs for SDP-1 lipase in *Arabidopsis* with an overexpression of its gene in accumulation conditions, a storage of the protein in the peroxisome and then a delivery to LDs in degradation conditions [68]. Both possibilities imply a transfer mechanism from the cytosol or an organelle to the LDs. Cytosolic proteins can associate transiently to LDs through structural features such as proline rich regions and amphipathic helixes, or after post-translational modifications such as acylation or prenylation [64,69].

**Figure 4:**
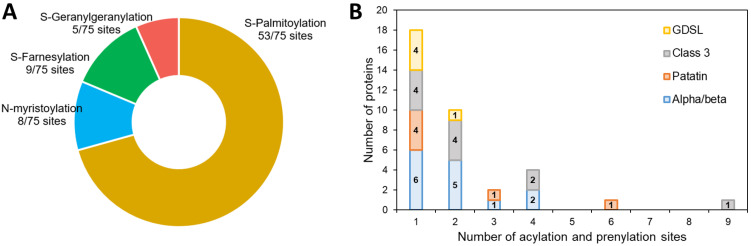
Prediction of acylation and prenylation sites of the candidate lipases. (**A**): proportion of each kind of modification in predicted sites, (**B**): number of modifications per protein distribution.

In our dataset, a prediction of acylation and prenylation sites using GPS-lipid shows that 36 out of 57 proteins have at least one site for these modifications (Table S4, Figure 4): 70% are sites of S-palmitoylation (Figure 4A), a modification known to enhance the hydrophobic character of a protein allowing better interactions with membranes [70]. This could be an important mechanism of protein-LD interaction during lipid catabolism. All the four families of proteins include predicted sites for such a modification, but two proteins have more than four predicted sites: the patatin-like lipase Phatr3_J46193 (six sites) and the class 3 lipase Phatr3_J47614 (nine sites) (Figure 4B). The latter was also one of the proteins with a slightly positive (hydrophobic) GRAVY score, suggesting a possible interaction with membranes and/or LDs.

Along with structural features, another possibility for the transfer of proteins between compartments could be the establishment of membrane contact sites as it occurs for SDP-1 lipase in *Arabidopsis*: SDP-1 is transferred from the peroxisomes to the LDs [68]. Close contact between LDs and peroxisomes and mitochondria have also been observed in the green microalgae *Micrasterias denticulata* and *Chlamydomonas reinhardtii* [71,72]. Moreover, membrane contact sites between LDs and other organelles have been reported to allow the transfer of lipids and other reaction products between compartments [64]. In diatoms, the β-oxidation of fatty acids occurs mainly in mitochondria and possibly in peroxisomes [73] so lipases assigned to these organelles could have access to TAG molecules through membrane contact sites. 

To explain the apparent absence of lipases from LDs proteomes obtained during nitrogen starvation, it can also be hypothesized that a large share of lipid consumption occurs by lipophagy. This process uses the mechanisms of autophagy to consume LDs by translocating them to the vacuole where lipases degrade the TAGs they contain. In this hypothesis, active lipases would be found mainly in the vacuole. However, protein targeting to the vacuole is not well-characterized in diatoms. It seems that a di-leucine based motif ([D/E]-XXXL-[L/I] or DXXLL) is necessary along with a signal peptide [74]. Here, only one α/β hydrolase (Phatr3_J15323), one class 3 lipases (Phatr3_J41624) and one patatin-like lipase (Phatr3_J45660) were predicted to be ER targeted (Figure 3). Hence, they could be located further in the secretory pathway, including Golgi, vacuole, plasma membrane or extracellular space localization. Inspection of the sequences from these three proteins indicates they contain di-leucine motifs and could hence be lipases acting in the process of lipophagy.

### 2.5. Transcription Data Reveal Contrasting Regulation across All Candidate Lipases

It is well-known that nitrogen starvation induces TAG accumulation in diatoms, mainly through a recycling of polar lipids [75], however the role of TAG lipases in this accumulation process is mostly unknown. As their main function is to degrade TAG, expression of these proteins is expected to be downregulated during a nitrogen stress, and to be upregulated when nitrogen is resupplied. 

To identify in our dataset which candidate lipases are responsive to stress conditions, the regulation of their transcripts, expressed as a log2 value, was retrieved from three experiments that specifically investigated the response of the whole cell to nitrogen starvation over time [75,76,77]. The three datasets exposed *P. tricornutum* cultures to a shift from standard nitrate-containing f/2 medium to nitrate deprived f/2. Those of Matthijs et al. (2016) [77] and Alipanah et al. (2015) [76] describe the response of the Pt1 ecotype (CCAP 1055/1) to a nitrogen stress in batch cultures during respectively 4 to 20 h and 48 to 72 h. The transcriptomic dataset of Scarsini et al. (2022) [75] monitors the response of the Pt4 ecotype (UTEX 646) during 15 days in a progressive transition to nitrogen deprivation. 

**Figure 5:**
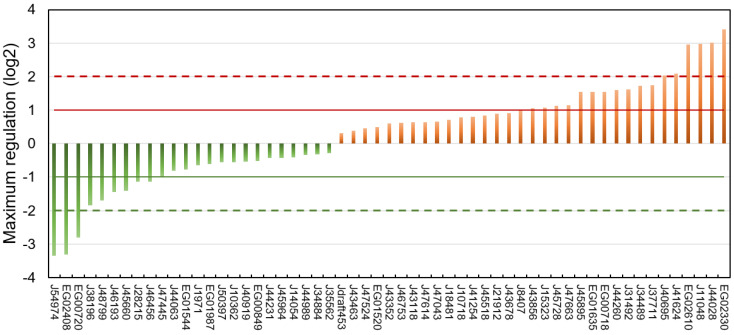
Maximum regulation expressed as log2 compared to a non-stressed situation observed across datasets [75,76,77] and all time points for each of the putative lipases.

The regulation of each putative lipase at every time point of the three experiments is reported in Table S5, and the highest regulation level observed over all datasets is shown in Figure 5.

Of the 57 putative lipases, 24 were downregulated and 33 upregulated. Considering a threshold of absolute log2 fold change of 1, 9 lipases were downregulated and 17 upregulated. 

Downregulated putative lipases during a nitrogen deficiency might be involved in the catabolism of neutral lipids stored in LDs during a nitrogen resupply. Among these lipases, the CGI-58 homolog Phatr3_J54974 (log2 −3.35) and the possible vacuolar lipase Phatr3_J45660 (log2 −1.41) can be cited. The patatin-lipase Phatr3_46193, for which an homologue has a galactolipase activity in *Pseudo-nitzschia* sp. [78], was also down-regulated (log2 −1.44). This is surprising because it has been previously described that during a nitrogen starvation in *P. tricornutum*, galactolipids are degraded and their acyl chains transferred to newly formed TAG [55,75]. 

It seems paradoxical that some putative TAG lipases are upregulated during neutral lipid storage. However, it has already been reported that lipases can also hydrolyze other substrates such as polar lipids from which acyl chains can be incorporated into newly formed neutral lipids in a process of recycling [55,75,79]. Indeed Nomaguchi et al. (2018) have found in the diatom *Fistulifera solaris* that some lipases are upregulated, while others are downregulated under lipid degradation conditions [52]. Upregulated candidate lipases include the plastid-located Phatr3_J44028 (log2 3.02) and the other possible vacuolar lipases Phatr3_J41624 (log2 2.09) and Phatr3_J15323 (log2 1.02). Surprisingly, upregulated putative lipases also include the lipase OmTGL (Phatr3_J37711, log2 1.75) previously reported to hydrolyze TAG enriched in EPA and for which a knockdown enhanced TAG content in the cell [39].

Overall, only nine proteins showed a maximum regulation of their transcripts higher than an absolute log2 > 2 (Table 1). These highly regulated lipases were chosen as the most plausibly involved in lipid metabolic processes and hence selected for the modelling of their 3D structure. 

### 2.6. Tertiary Structure Prediction of the Most Regulated Candidate Lipases Reveals a Functional Lipase Fold and Additional Domains That May Regulate Catalytic Activity

**Figure 6:**
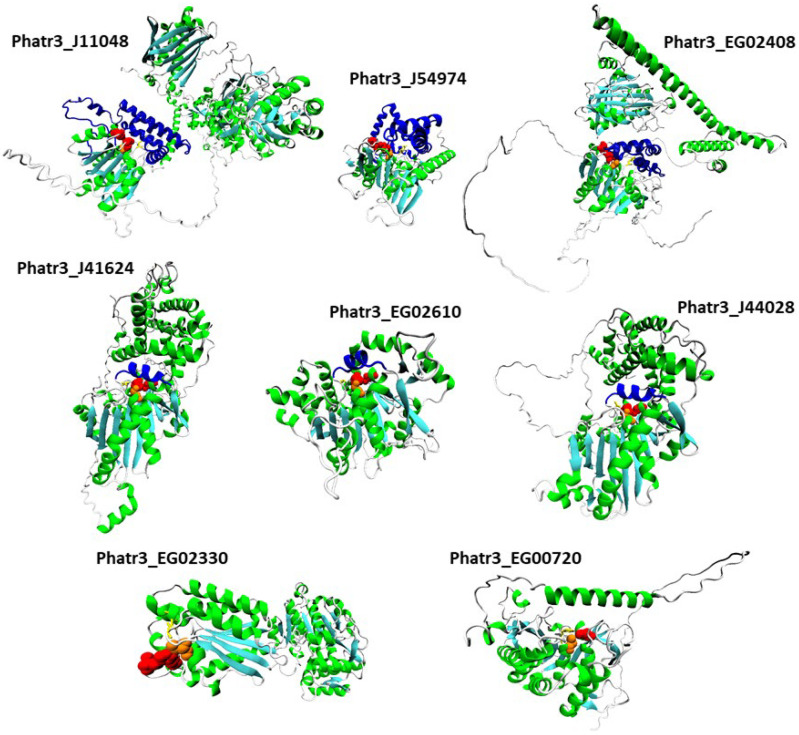
Tertiary structure models of the eight most regulated candidate lipases. Alpha helixes are displayed in green while beta sheets are in cyan. Catalytic residues are displayed with a van der Waals representation in orange for the catalytic serines and red for other residues. Oxyanion hole residues are displayed with a licorice representation in yellow. Lids and caps are displayed in blue.

For each protein sequence, the Alphafold algorithm was run twice on Colabfold, once with a search for templates for modelling, once without. For each protein, the best model of the two runs was retained according to Colabfold internal metric (predicted local Distance Difference Test, plDDT). The positions of catalytic and possible oxyanion hole residues were investigated, and structures are reported in Figure 6. Structure inspection of the upregulated α/β hydrolase Phatr3_J40695 revealed that its pentapeptide GVSHG motif was outside of the α/β hydrolase fold domain, and that no potentially catalytic histidine could be found in a localization near the serine. Hence, it can be considered that it is not a lipase, and this protein was excluded from further analysis.

All the remaining proteins had a similar structure with a β sheet composed of β strands (in cyan in Figure 6) separated by α helixes (in green in Figure 6). Class 3 lipases and α/β hydrolase shared the classic α/β fold, with eight β strands and six α helixes and the conserved pentapeptide (including the catalytic serine) forming a nucleophilic elbow structure at the center of the fold, in a junction between a β strand and the following α helix [33]. Patatin-like lipase Phatr3_EG00720 displays a similar 3D arrangement of its pentapeptide but it is inside a β sheet made of only six β strands as previously described for this family of proteins [49]. For the GDSL lipase Phatr3_EG02330, the structure is also similar to other GDSL lipases with five β strands [80]. In all families, the catalytic and oxyanion hole residues were all grouped in one well-defined catalytic site located approximately at the center of the β sheet. For the patatin-like Phatr3_EG00720, as observed in the sequence alignment (Figure 1C), the oxyanion hole motif was different from that of other members of its family. However, one proline residue found near this position (661 in the alignment, Figure 1C) could serve as a stabilizing residue by presenting its backbone to the active site. Indeed, unusual but functional oxyanion hole residues exist within α/β hydrolases such as a Serine-Isoleucine motif for a hydrolase of the bacteria *Pseudomonas aeruginosa* PA01 or a Proline-Phenylalanine motif for a N-acyl homoserine lactone degrading enzyme of the same strain [34]. Hence, all structures were plausible and consistent with previous structural data.

Alphafold provides a confidence score for the models, the predicted local Distance Difference Test (plDDT) that is an indicator of whether the structure should be trusted. For all structures, the average plDDT is higher than 70 indicating good overall confidence in all the eight generated 3D models. However, the score is variable along the structure. Globally, the conserved domain parts are predicted with high confidence (80–90), confirming the observed folds. However, some structures such as that of Phatr3_EG02408 or that of Phatr3_J11048 include poorly predicted domains (<50), especially extended *N*-terminal strands and sequences separating two well-defined domains. 

**Figure 7:**
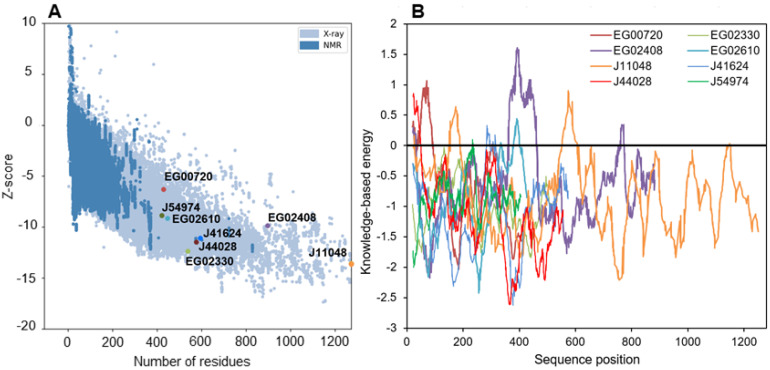
Energy characteristics for all the modelled structures as calculated by ProSa. (**A**): Z-score of structures as displayed within the range of experimentally determined structures, (**B**): knowledge-based energy plot averaged over 40 residues along the structures.

The quality of the structures was further evaluated by tracing Ramachandran diagrams that are a plot tracing the two torsional angles around the Cα of each amino acid in a protein structure. Due to steric hindrance between side chains, not angle all values can be adopted and the diagram defines regions that are energetically favored, allowed or unlikely, termed respectively “highly favored”, “favored” and “questionable” in the Zlab server. This analysis conducted on the full predicted structures showed that four proteins had more than 90% of their amino acids in highly favored regions, while the four remaining only had between 80 and 90% of such residues and a high share (8.4 to 13.7%) of amino acid residues in a “questionable” position (Table S6). Removing the poorly predicted (according to plDDT) extended strands from the models nearly suppressed amino acids in those regions, in agreement with plDDT score (Table S6). Two other metrics were calculated to further evaluate the quality of the structures (Figure 7). The Z-score is an indicator of how the total folding energy of the structure compares to the energy of a random arrangement of the atoms. Figure 7A shows that all models are within the range of experimentally determined protein structures of the same length, as expected for valid structures. Energy was also traced for each amino acids in the sequence (rolling average over 40 residues, Figure 7B) to investigate local model quality. As this metric is generally negative in native structures, positive values highlight poor quality parts of the 3D models [81]. As for plDDT scores, the domains showing positive value of energy are either *N*-terminal uncertain domains or linkage sequence between two well-predicted domains.

It is especially the case for the downregulated Phatr3_EG02408, which clearly displays two domains separated by a long poorly predicted sequence of approximately 300 amino acids (Figure 6 and Figure 7B). The gene encoding this protein was created in the latest genome annotation of *P. tricornutum* by merging two genes (Phatr2_31407 and Phatr2_31408) from the previous annotation, a process carried out when two closely located genes are regulated similarly in transcriptomics data and encode protein domains often found together in other known-proteins [48]. The dataset by Matthijs et al. (2016) [77] was using the Phatr2 annotation and both genes showed different regulation levels, the Phatr2_31408 part being much more downregulated than the Phatr2_31407 one (at 8 h of nitrate starvation, a log2 of −3.2 against −0.53). This variation in regulation for two parts of the same gene, along with the poorly predicted linkage sequence between the two protein domains highlight that this gene model is uncertain and should be further investigated. However, if this annotation is correct, the first domain would be the classic α/β fold, while the second one corresponds to a “polyketide cyclase/dehydrase and lipid transport” superfamily domain. This second domain is present in a vast protein family including members that act in non-vesicular lipid transport through the binding of different lipid species including cholesterol and phosphatidyl-choline [82]. As it was predicted to be located in the plastid in our analysis, Phatr3_EG02408 could participate in lipid exchange between plastid membranes or between other organelles membranes and plastid membranes. Another α/β hydrolase, Phatr3_J11048, also had different domains, including a *C*-terminal enolase domain, and a possible “polyketide cyclase/dehydrase and lipid transport” domain. The presence of three different functional domains suggests a complex functioning for this protein that should be investigated by functional genomics and/or purification. 

In addition to the α/β fold, it is common for lipases structures to include one or several additional domains that cover and control the access of substrates to the catalytic site [34]. By inspection of our 3D models, the three class 3 lipases were found to share a common structure above the catalytic site made of one helix (in blue in Figure 6). This helix could be a lid, a mobile domain that can take two conformations: one open that lets the substrate enter the active site, and one closed blocking it. Lids are crucial to the interfacial activation of lipases and allow a high activity when in contact with lipids [33], at the surface of lipid droplets for instance. 

In our three α/β hydrolase models including the CGI-58 homolog (Phatr3_J54974), another type of structure made of five α helixes was found above and around the catalytic site (Figure 6, Figure S1). These structures are possibly caps, a kind of domain that also covers the catalytic site like lids, but that are immobile and where the substrate passes through a tunnel to reach the active residues. Such a cap domain is also present in the human CGI-58 experimental structure [83]. Both the patatin-like lipase (Phatr3_EG00720) and the GDSL lipase (Phatr3_EG02330) have an active site directly accessible from the surface which could mean a different type of activity. Along with functions in plant growth and development, GDSL lipases are also known as regulators of plant response to stress [84]. Currently, no GDSL lipase from microalgae has been characterized, so their function in these organisms is unknown. As it is highly regulated under nitrogen starvation, the GDSL lipase Phatr3_EG02330 could be a driver of the response of diatoms to stress. Indeed, the predicted tertiary structure of this protein shows that the canonical GDSL domain is followed by a NADP-binding domain that could be involved in regulation mechanisms, as nutrient stress conditions induce an imbalance in redox status in the cell [69].

## 3. Materials and Methods

### 3.1. Steps for the Screening Procedure and Retrieval of Sequences

The latest *P. tricornutum* genome annotation is termed Phatr3 [48] and is available at the Ensembl portal (http://protists.ensembl.org/Phaeodactylum_tricornutum/Info/Index, accessed on 2 May 2022). The annotation was queried with the research terms “lipase” and “phospholipase”. Lipases are hydrolases with a α/β hydrolase domain, and some proteins annotated as such could be lipases, as is the case for the CGI-58 homolog Phatr3_J54974. Hence, the term “alpha/beta hydrolase” was also searched. Other proteins identified as lipases in previous studies were also included: a patatin-like phospholipase domain containing protein 3 (Phatr3_J46511) [85] and two phospholipase A proteins (Phatr3_J44005 and Phatr3_J44066) [27]. Full length amino acid sequences were then downloaded from Ensembl. Sequences were first submitted in the Pfam database (http://pfam-legacy.xfam.org/, accessed on 1 June 2022), and a threshold E-value of 10^−5^ was used to consider a hit as a significant annotation. Then, as lipases should include a GXSXG motif (excepting GDSL lipases), sequences not containing this motif were excluded from the dataset. Finally, as α/β hydrolases can have a range of hydrolytic activities (not necessarily toward lipids), proteins annotated as such were submitted to the ESTHER database (https://bioweb.supagro.inrae.fr/ESTHER/general?what=index, accessed on 1 June 2022). Enzymes for which the best hit was annotated with a precise function (i.e., proline iminopeptidase, pheophytinase…) other than lipase were excluded from the analysis.

### 3.2. Multiple Alignment of Sequences

Sequences from the identified proteins with a possible TAG lipase function were aligned using the software MAFFT (Multiple Alignment Program for Amino Acid or Nucleotide Sequences) with default parameters (MAFFT flavor auto, Gap extension penalty 0.123, Gap opening penalty 1.53, No matrix) through the NG phylogeny platform (https://ngphylogeny.fr/, accessed on 3 June 2022). 

### 3.3. Determination of Physico-Chemical Characteristics

Molecular weight, number of positively and negatively charged amino acids, extinction coefficient at 280 nm in water, isoelectric point, half-life of the protein, instability index, aliphatic index and Grand Average of Hydropathy (GRAVY) were obtained using the ProtParam tool available online at Expasy (http://web.expasy.org/protparam/, accessed on 24 August 2022).

### 3.4. Subcellular Localization Prediction and Post-Translational Modification

In order to predict the subcellular location of proteins, an adapted version of the pipeline proposed by Van Tol et Armbrurst (2021) [86] was used (Figure S2). The candidate sequences were used as inputs for TargetP 2.0 [87], SignalP 6.0 [88], SignalP 3.0 [89], HECTAR [90], ASAFind [91] and MitoprotII [92]. The ScanProsite tool (https://prosite.expasy.org/scanprosite/, accessed on 24 August 2022) was also run to search for a potential ER-retention signal [K/D]-[D/E]-E-L and for peroxisome targeting signal 1 and 2, respectively [S/A/C]-[K/R/H]-[L/M] and S-S-L, at the end of the *C*-terminus of the protein. Additionally, DeepTMHMM [93] was used to predict transmembrane domains. Seven possible locations can be predicted through this pipeline: cytosol, ER/cytosol, ER, chloroplast, mitochondria, mitochondria/peroxisome, and peroxisome. Potential sites for post-translational acylation and prenylation were predicted using GPS-Lipid [94] with the high stringency option. Vacuolar di-leucine targeting signals ([D/E]-XXXL-[L/I] or DXXLL) were searched with ScanProsite. 

### 3.5. Tertiary Structure Prediction and Quality Analysis

The translated amino acid sequence of the most regulated candidate proteins were used as inputs for tertiary structure prediction by Alphafold2 through the Colabfold interface with the “template_mode” option set as pdb70 (https://colab.research.google.com/github/sokrypton/ColabFold/blob/main/AlphaFold2.ipynb, accessed on 29 August 2022) [95]. Alphafold2 is the first algorithm that predicts protein tertiary structure to a precision close to experimental models [96]. It is based on deep neural networks trained with structures available on the Protein Database (PDB). Briefly, the algorithm searches for homologues of the primary sequence in databases and generates a multiple sequence alignment (MSA). In the case of Colabfold, the database is a combination of BFD and MGnify databases expanded using metagenomics sequences from different environment and domains of life [95]. The primary sequence is also used to retrieve the 20 best structure templates in the PDB70, a clustered version of the PDB. Both MSA and templates found are used as input for the algorithm which predicts the 3D coordinates of amino acids backbones and residues. For refining the 3D architecture, the obtained structure is fed back into the algorithm 3 times (recycle count = 3). Alphafold2 produces 5 models using different random seeds and the use or not of templates. The models are then compared using a predicted local Distance Difference Test (plDDT) which is an assessment of the confidence one can have in the predicted structure. The model with the highest mean plDDT is considered the best structure. For interpretation, a plDDT > 70 is considered to be confident (90 being very confident), while under 50 the region should be considered as random or disorganized [96]. VMD 1.9.3 was used to visualize the structures and to highlight residues. Ramachandran plots were traced in Z-lab (https://zlab.umassmed.edu/bu/rama/, accessed on 30 August 2022). Energy scores of the structures were obtained using the Protein Structure Analysis-web tool (https://prosa.services.came.sbg.ac.at/prosa.php, accessed on 2 September 2022) [81] and data extracted using WebPlotDigitizer (https://automeris.io/WebPlotDigitizer/, accessed on 2 September 2022).

## 4. Conclusions

The aim of this study was to use an in silico approach to identify relevant enzymes playing roles in the lipid catabolism in *P. tricornutum*. Our screening approach yielded 57 candidate lipases in 4 families: α/β hydrolases, class 3 lipases, patatin-like phospholipases and GDSL lipases. Further in silico investigations of these putative enzymes highlighted 8 highly regulated enzymes that could hence be active in lipid metabolism response to a nitrogen availability shift, and four others (Phatr3_J46193, Phatr3_J47614, Phatr3_J45660 and Phatr3_J15323) that were less regulated but showed interesting features in their predicted subcellular localization.

General characterization of the set of enzymes revealed that a majority of them was hydrophilic and located to the cytosol, suggesting that they could temporarily be associated with LDs from the cytosol in a process of lipolysis. Three enzymes, Phatr3_J15323, Phatr3_J41624 and Phatr3_J45660 were predicted to possibly be vacuolar, suggesting they could act in lipophagy, the second hypothesized pathway for lipid degradation in diatoms. The respective contribution of those two mechanisms to lipid catabolism in diatoms is not known and could be investigated using inhibitors of lipase activity and of autophagy.

Transcription patterns highlighted that candidate lipases could be regulated in both directions (up and down) as a response to nitrogen stress. Enzymes that are downregulated during lipid accumulation are promising candidates for a TAG lipase activity. The most downregulated lipase is the CGI-58 homolog Phatr3_J54974 which inactivation has already been shown to enhance TAG accumulation and could hence be a major lipolysis actor. Two other proteins were highly downregulated and could hence be relevant actors in the lipolysis process: the α/β hydrolase Phatr3_EG02408, and the patatin-like Phatr3_EG00720 which transmembrane helix could permit to translocate from the ER to LDs. The 3D modelling showed that all three candidates had well-predicted lipase structures while Phatr3_EG02408 was possibly made of two distinct proteins. 

Further work should then use Phatr3_J54974 and Phatr3_EG00720 as targets for overexpression, knockdown or knockout in order to investigate mechanisms of lipolysis and to improve lipid production. Purification should also be conducted to characterize their activity on various lipidic substrates and search for new enzymatic activities of biotechnological interest. 

## Figures and Tables

**Table 1:** Characteristics of the nine candidate lipases showing the highest regulation.

Protein ID (Phatr3)	Family	Regulation (log2)	GRAVY	Predicted Localization
J54974	α/β	−3.35	−0.35	Cytosol
EG02408	α/β	−3.32	−0.091	Chloroplast
EG00720	Patatin	−2.80	0.078	ER/Cytosol
J40695	α/β	2.03	−0.108	Cytosol
J41624	Class 3	2.09	−0.411	ER and/or vacuole
EG02610	Class 3	2.96	−0.357	Cytosol
J11048	α/β	2.98	−0.304	Cytosol
J44028	Class 3	3.02	−0.381	Chloroplast
EG02330	GDSL	3.41	−0.168	Cytosol

## Data Availability

The original data presented in the study are included in the article/Supplementary Materials; further inquiries can be directed to the corresponding author.

## Supplementary Materials

The following supporting information can be downloaded at: https://www.mdpi.com/article/10.3390/md21020125/s1, Figure S1: Alternative view of the cap domain (blue) of the CGI-58 homolog Phatr3_J54974 that surround the catalytic site; Figure S2: Pipeline used for the prediction of subcellular localization; Table S1: List of retrieved sequences through a search in the genome annotation of *Phaeodactylum tricornutum* Phatr3 (Rastogi et al. 2018) and screening through the pfam and ESTHER databases; Table S2: Protparam complete results obtained from the Expasy server; Table S3: Sub-cellular prediction for putative lipase localization; Table S4: Prediction of prenylation and acylation sites by GPS lipids; Table S5: Complete transcription data for the 57 lipases identified in this study; Table S6: Ramachandran analysis results as obtained from the Z-lab server.
