# Supplementary figures and images for: Bioinformatics-Based Screening Approach for the Identification and Characterization of Lipolytic Enzymes from the Marine Diatom Phaeodactylum tricornutum
Source: Mar Drugs. 2023 Feb 14;21(2):125. doi: 10.3390/md21020125 (PMC9964374; doi:10.3390/md21020125)

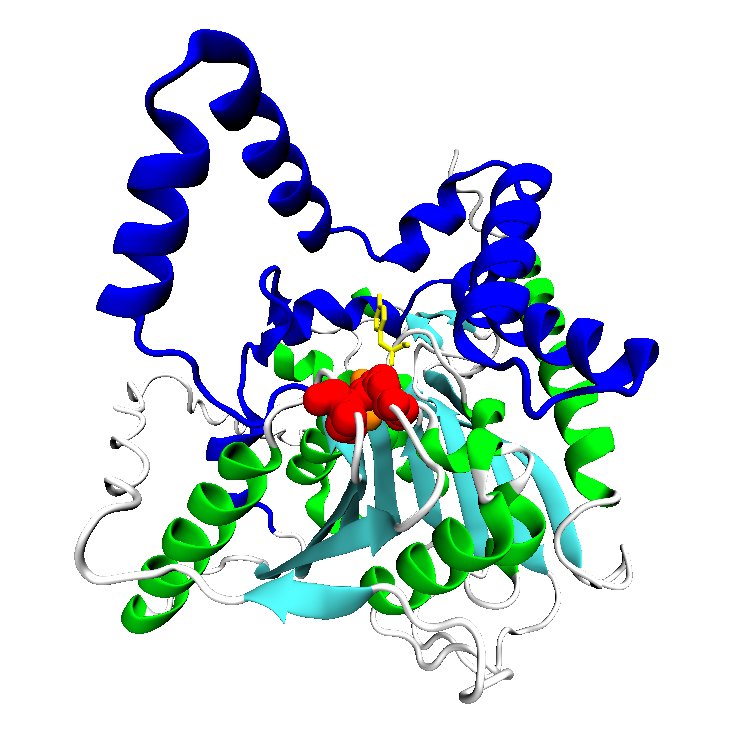

Supplement: Supplementary file 1 [file marinedrugs-21-00125-s001.zip › Figure S1.png]

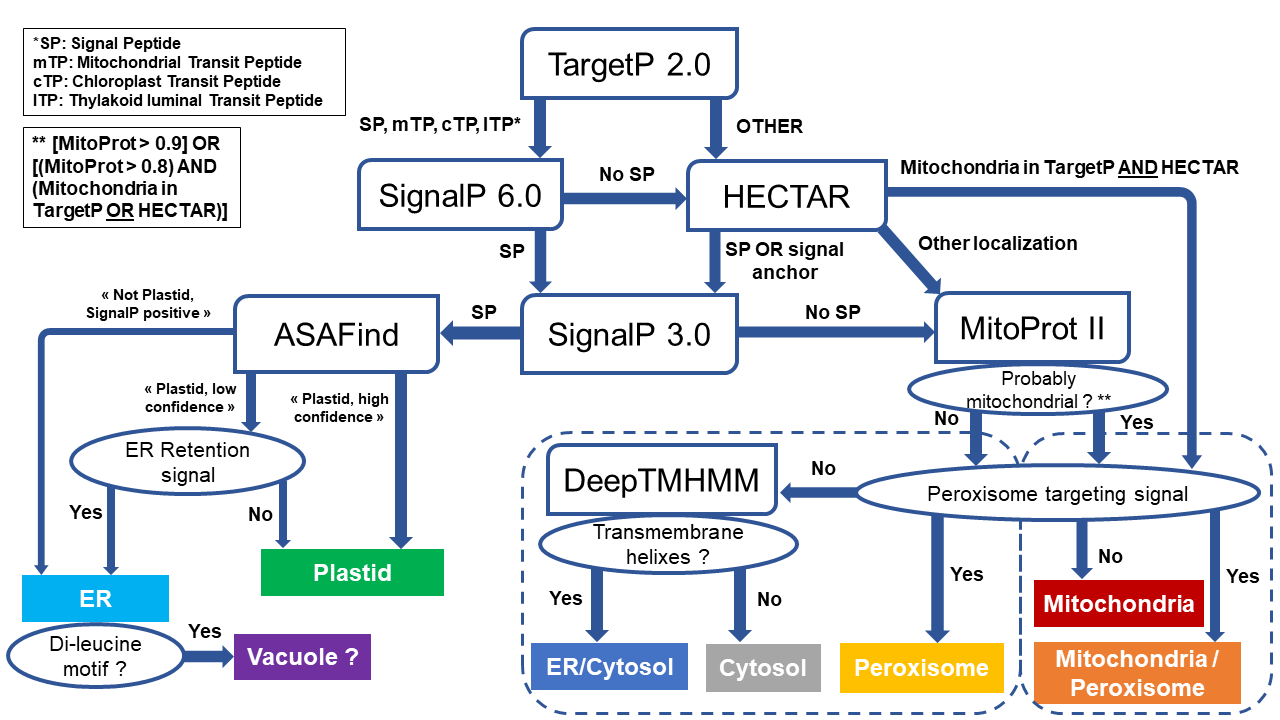

Supplement: Supplementary file 1 [file marinedrugs-21-00125-s001.zip › Figure S2.png]
